# Etomoxir, a carnitine palmitoyltransferase 1 inhibitor, combined with temozolomide reduces stemness and invasiveness in patient-derived glioblastoma tumorspheres

**DOI:** 10.1186/s12935-022-02731-7

**Published:** 2022-10-11

**Authors:** Jin-Kyoung Shim, Seonah Choi, Seon-Jin Yoon, Ran Joo Choi, Junseong Park, Eun Hee Lee, Hye Joung Cho, Suji Lee, Wan-Yee Teo, Ju Hyung Moon, Hyun Sil Kim, Eui Hyun Kim, Jae-Ho Cheong, Jong Hee Chang, Jong In Yook, Seok-Gu Kang

**Affiliations:** 1 Department of Neurosurgery, Brain Tumor Center, Severance Hospital, Yonsei University College of Medicine, Seoul, 03722 Republic of Korea; 2 Brain Tumor Translational Research Laboratory, Severance Biomedical Research Institute, Yonsei University College of Medicine, Seoul, 03722 Republic of Korea; 3 Precision Medicine Research Center, College of Medicine, The Catholic University of Korea, Seoul, 03722 Republic of Korea; 4 Department of Medical Science, BK21 Plus Project for Medical Science, Yonsei University College of Medicine, Seoul, 03722 Republic of Korea; 5 Cancer and Stem Cell Biology Program, Duke-NUS Medical School, Singapore, 169857 Singapore; 6 Institute of Molecular and Cell Biology, A*STAR, Singapore, 169857 Singapore; 7 Department of Oral Pathology, Yonsei University College of Dentistry, Seoul, 03722 Republic of Korea; 8 Department of Surgery, BK21 Plus Project for Medical Science, Yonsei University College of Medicine, Seoul, 03722 Republic of Korea; 9 Departments of Medical Science, Yonsei University Graduate School, Seoul, 03722 Republic of Korea

**Keywords:** Etomoxir, Fatty acid oxidation, Glioblastoma, Temozolomide, Tumorsphere

## Abstract

**Introduction:**

The importance of fatty acid oxidation (FAO) in the bioenergetics of glioblastoma (GBM) is being realized. Etomoxir (ETO), a carnitine palmitoyltransferase 1 (CPT1) inhibitor exerts cytotoxic effects in GBM, which involve interrupting the FAO pathway. We hypothesized that FAO inhibition could affect the outcomes of current standard temozolomide (TMZ) chemotherapy against GBM.

**Methods:**

The FAO-related gene expression was compared between GBM and the tumor-free cortex. Using four different GBM tumorspheres (TSs), the effects of ETO and/or TMZ was analyzed on cell viability, tricarboxylate (TCA) cycle intermediates and adenosine triphosphate (ATP) production to assess metabolic changes. Alterations in tumor stemness, invasiveness, and associated transcriptional changes were also measured. Mouse orthotopic xenograft model was used to elucidate the combinatory effect of TMZ and ETO.

**Results:**

GBM tissues exhibited overexpression of FAO-related genes, especially *CPT1A*, compared to the tumor-free cortex. The combined use of ETO and TMZ further inhibited TCA cycle and ATP production than single uses. This combination treatment showed superior suppression effects compared to treatment with individual agents on the viability, stemness, and invasiveness of GBM TSs, as well as better downregulation of FAO-related gene expression. The results of in vivo study showed prolonged survival outcomes in the combination treatment group.

**Conclusion:**

ETO, an FAO inhibitor, causes a lethal energy reduction in the GBM TSs. When used in combination with TMZ, ETO effectively reduces GBM cell stemness and invasiveness and further improves survival. These results suggest a potential novel treatment option for GBM.

**Supplementary Information:**

The online version contains supplementary material available at 10.1186/s12935-022-02731-7.

## Introduction

Glioblastoma (GBM), which is now newly defined as an IDH-wildtype tumor only [1], is a primary malignant brain tumor with high mortality [2]. Although the Stupp regimen settled down as a standard treatment, the median survival is still about 15–20 months [2, 3]. Despite extensive studies performed on GBM, little is known about its therapeutic strategies [4, 5]. Interventions targeting cancer cell metabolism have been considered one of the potential key strategies to overcome drug resistance [6]. Previously, we demonstrated positive therapeutic efficacy on GBM TSs by inhibiting oxidative phosphorylation (OxPhos), a mitochondrial electron transport chain (ETC) that produces most ATPs in cells [7–10]. Our results provided evidence that ATP depletion leads to the decline of stemness and invasiveness, thereby increasing patient survival.

Fatty acids have higher NADH, NADPH, and FADH_2_ production rates per unit than glucose [11]. These molecules are involved in ATP production, anabolic processes, and antioxidant activity, which regulate cell growth and proliferation. Several types of tumors, such as leukemia [12], prostate [13], and breast cancer [14], utilize fatty acid oxidation (FAO) for energy production. This phenomenon has also been reported in glioma [15, 16]. ETO irreversibly inhibits CPT1, which is a gateway of fatty acid transfer through the mitochondrial membrane, and blocks the initiation of FAO [15–17]. One recent research revealed that patient-derived GBM cells express FAO-related enzymes and rely on FAO for aerobic respiration and proliferation, thereby ETO prolongs survival in a mouse model [15].

TMZ had been as a standard chemotherapeutic agent in GBM treatment [2] for decades, and there are currently no alternatives or combinations that achieve better results than TMZ. We therefore attempted to elucidate the combinatory effects of TMZ and ETO, which have not been reported previously. The therapeutic responses to ETO and/or TMZ were evaluated using patient derived GBM TSs and orthotopic xenograft mouse models.

## Materials and methods

### Processing of transcriptome data

In total, 112 IDH-wildtype GBM tissues and 35 tumor-free cortex tissues were obtained from 138 glioma patients who had undergone surgical resection at the Yonsei University Severance Hospital, Seoul, Republic of Korea. All GBM cases with sufficient tissue amount for RNA sequencing were included in this study, and there was no other exclusion criteria. Tumor-free cortex tissues were gained randomly among glioma patients. Total RNA was extracted from the tissues or GBM TSs for use in RNA sequencing. Gene expression was measured with Cufflinks v2.1.1 [18] using gene sets published by Mootha et al. [19] and the gene annotation database Ensembl (release 72) [20]. Gene set variation analysis (GSVA) scores were used to evaluate FAO-related genes, and the average linkage of hierarchical clustering for GBM TSs was calculated using Pearson's correlation coefficient as a distance metric. Expression levels are shown as heatmaps using the GENE-E software.

### GBM TS culture and reagents

GBM TSs were derived from four different fresh IDH-wildtype GBM tissue specimens as previously described [9, 21]. The specimens were denoted as TS15-88, TS14-15, TS14-08, and TS13-64. GBM TSs were grown in TS complete medium consisting of DMEM/F-12 (Mediatech, Manassas, VA, USA), 1x B27 (Invitrogen, San Diego, CA, USA), 20 ng/mL basic fibroblast growth factor (bFGF; Novoprotein, Summit, NJ, USA) and 20 ng/mL epidermal growth factor (EGF; Novoprotein). This medium was used in all in vitro experiments. ETO (Sigma-Aldrich, St. Louis, MO, USA) and TMZ (Sigma-Aldrich) were dissolved in dimethyl sulfoxide and used at 100 μM and 250 μM, respectively. ETO dose was decided through a combination index plot with the best synergism with TMZ.

### ATP level and cell viability assay

The effects of the ETO and/or TMZ on GBM TS survival were determined using luciferase and WST assays. The assays were performed as previously described [7, 22]. CompuSyn software was used to determine the combination indices (CIs) of ETO and TMZ therapy under Chou-Talalay method [22].

### Metabolite quantification using liquid chromatography-mass spectrometry

GBM TSs were treated with ETO and/or TMZ for 48 h. They were then harvested and extracted using cold methanol/ H_2_O (80/20, v/v). The samples were analyzed by LC–MS/MS using a 1290 HPLC Qtrap 5500 (ABSciex, Redwood City, CA, USA) equipped with a reverse phase column (Synergi Fusion-RP 50 × 2 mm) to examine the metabolites associated with energy metabolism [23].

### Measurement of cellular NADH/NADPH

To measure the NADPH levels, GBM TSs were treated with ETO and/or TMZ for 72 h using an NADP/NADPH Quantification Colorimetric Kit (BioVision, Milpitas, CA, USA). Their levels were quantified by determining the optical density values at OD450 nm using a VersaMax microplate reader (Molecular devices, Sunnyvale, Ca, USA).

### XF cell mitochondrial stress analysis

GBM TSs were treated with ETO and/or TMZ for 48 h. Their oxygen consumption rates (OCRs) were assessed using the Seahorse XF-24 instrument (Seahorse Bioscience, North Billerica, MA, USA) as previously described [7].

### Apoptosis

GBM TSs were treated with for 72 h with ETO and/or TMZ and dissociated with accutase (sigma-Aldrich). After, GBM TSs were incubated in binding buffer consisting of FITC-conjugated annexin V (BioLegend, CA, USA) and propidium iodide (PI, Sigma-Aldrich) for 10 min in the dark. Total 1 × 10^5^ cells were examined using an LSR II flow cytometer (BD Biosciences, NJ, USA).

### Western blot analysis

GBM TS lysates were separated using sodium dodecyl sulfate–polyacrylamide gel electrophoresis. Western blots were performed following previous description [9, 22]. The ImageQuant LAS 4000 mini was used to acquire images of the protein bands (GE Healthcare Life Sciences, Little Chalfont, UK). The original images of full-length blots were added as supplementary (Additional file 2: Fig. S5-7).

### Neurosphere formation assay

Ten single GBM cells were seeded in 96-well plates and maintained in TS complete media—for 3 weeks. The TS complete media were supplemented once a week. The percentage of sphere-positive wells was computed and displayed based on the control group. ToupView software was used to capture and analyze the images (ToupTek Photonics, Zhejiang, China).

### Three-dimensional (3D) invasion assay

A mixed matrix consisting of Matrigel, collagen type I (Corning, Tewksbury, MA, USA), and TS complete medium was placed in each well of a 96-well plate. Single spheroids were implanted into each matrix and awaited until they became gelatin form. Then, the TS complete media containing ETO and/or TMZ were applied to each well. The invaded area was measured as an occupied area at (72 h–0 h)/0 h.

### Mouse orthotopic xenograft models

In this experiment, male athymic nude mice (4–8 weeks old; Central Lab, Animal Inc., Seoul, Republic of Korea) were used. For the pretreatment step [24–29], GBM TSs (TS13-64) were pretreated for 3 days with ETO and/or TMZ. Trypan blue staining was performed to select dissociated TSs. Live cells (5 × 10^5^) were implanted into the right frontal lobes of the mice at a depth of 4.5 mm using a guide-screw system [30]. The mice were euthanized as per protocol, if their maximal body weight dropped by more than 15%. The mice brains were cut into 4 μm thickness using a microtome and fixed on adhesive slides. Immunohistochemistry (IHC) stain with Nestin and Zeb1 was done using a peroxidase/3,3ʹ-diaminobenzidine staining system.

### Bioluminescence imaging

Bioluminescence data of mouse models was collected and analyzed using IVIS imaging equipment and Living Image v4.2 software (Caliper Life Sciences, Hopkinton, MA, USA). Animals were injected intraperitoneally with 100 L D-luciferin (30 mg/mL; Promega) 15 min before signal acquisition.

### Statistical analysis

One-way ANOVA with Tukey’s post hoc test was conducted for multiple comparisons between the treatment groups. Survival rates was calculated with the Kaplan–Meier method and compared using log-rank tests. GraphPad Prism 7 (GraphPad Software Inc., San Diego, CA, USA) was used for all graphs and statistical analyses. Results with *P < 0.05, **P < 0.01, and ***P < 0.001 were considered statistically significant.

## Result

### GBM upregulates FAO

**Fig. 1:**
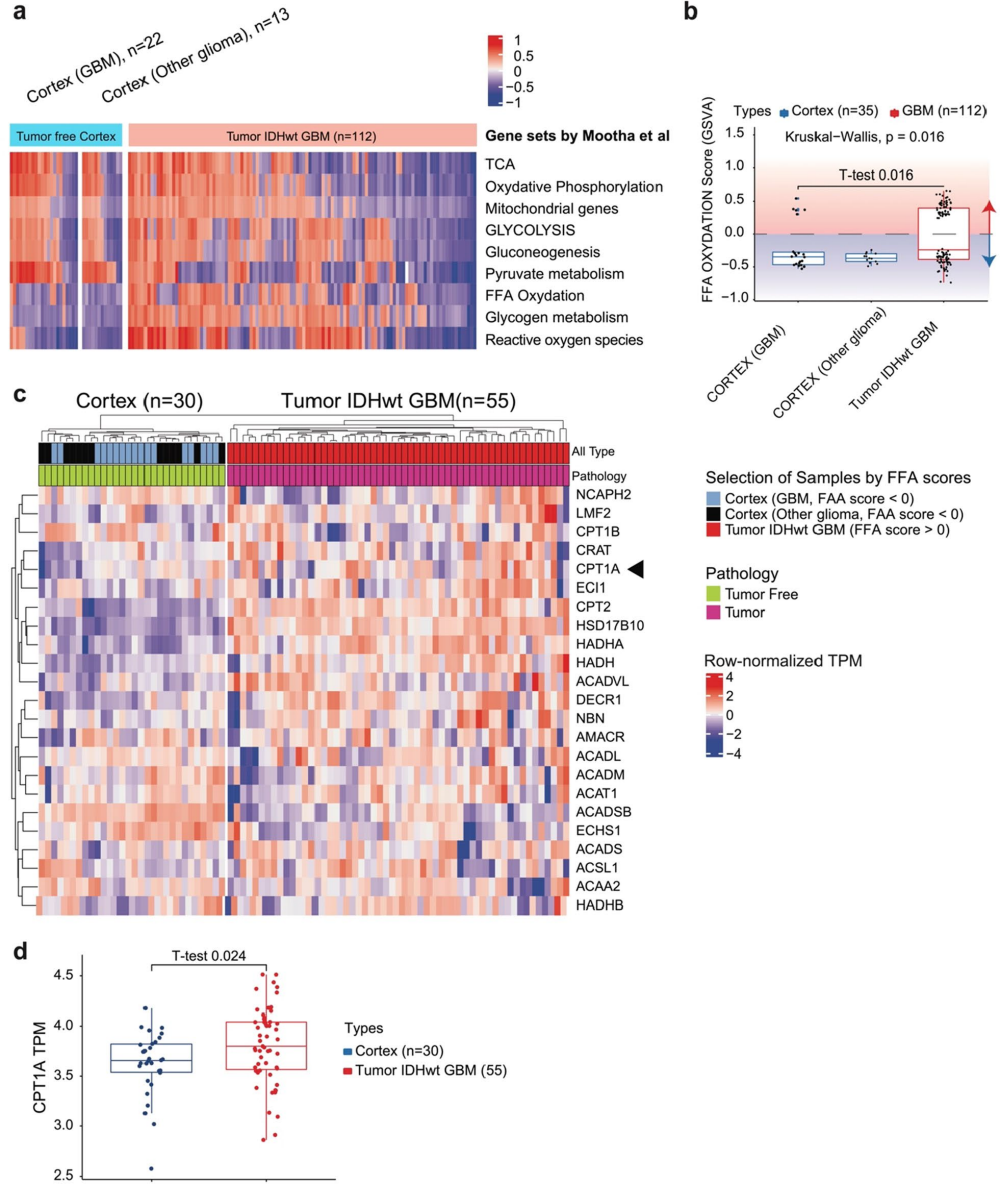
Summary of preceding research data using patient-derived glioma tumor samples. **a** Compared to the tumor-free cortex, IDH-wildtype GBM strongly presented upregulated gene sets related to FAO, glycogen metabolism, and reactive oxygen species while expression of genes associated with glycolysis was comparable between groups. **b** The FFA oxidation score by GSVA was much higher in IDH-wildtype GBM tumorspheres than the tumor-free cortex. **c** Expression of genes related to FAO was compared separately. Expression of *CPT1A* (black arrowhead) and *CPT2*, encoding CPT1 and CPT2, respectively, which are major pacemaker enzymes located in the outer and inner mitochondrial membranes that initiate FAO, was upregulated in GBM. **d** *t*-tests confirmed statistical significance of *CPT1A* differential expression between the cortex and tumor

Prior to performing the main study, we analyzed the differential expression of metabolism-related genes between the tumor-free cortex and GBM. The clinical and histopathological characteristics are listed in Additional file 1: Table S1. Among the metabolism-related gene sets, the expression of those involved in FAO, glycogen metabolism, and reactive oxygen species (ROS) was significantly upregulated in GBM (Fig. 1a). The GSVA scores showed relative overexpression of FAO-related genes in GBM (Fig. 1b). Among the FAO-related genes, expression levels of *CPT1A* and *CPT2* were upregulated (Fig. 1c). These genes encode the major pacemaker enzymes CPT1 and CPT2, respectively. In addition, *t*-tests confirmed a significant difference in *CPT1A* expression between the tumor-free cortex and the GBM (Fig. 1d).

### Combined treatment synergistically reduces the cellular viability of GBM TSs

**Fig. 2:**
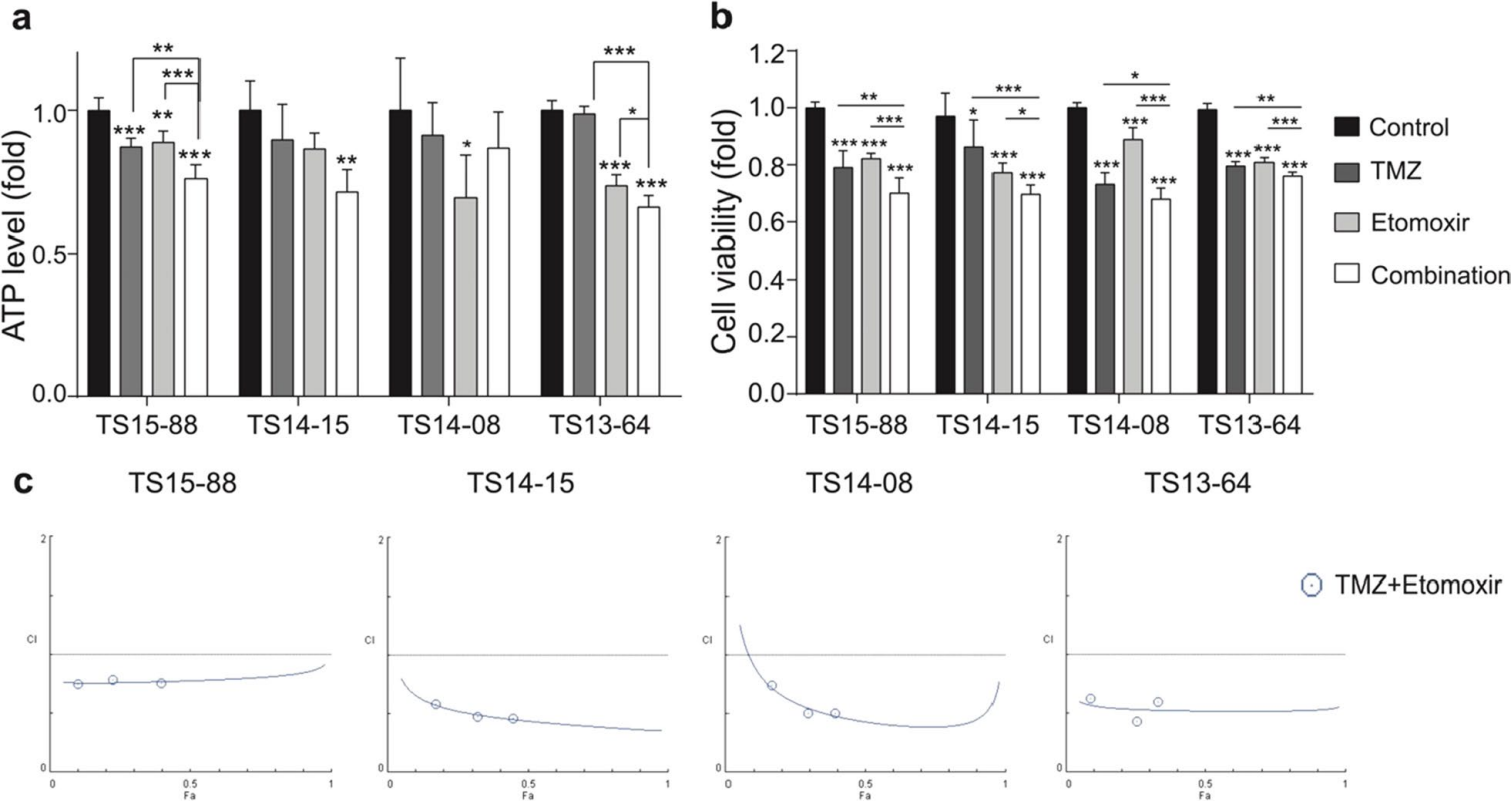
The effects of combined ETO and TMZ treatment on the viability of GBM TSs. **a** The ATP levels of GBM TSs were measured using a luciferase assay after treatment with ETO and/or TMZ. ATP levels decreased not only in ETO treatment but also in TMZ, however, the combination treatment surpassed individual treatments. **b** The cell viability of GBM TSs were measured using a WST assay after treatment with ETO and/or TMZ, and the same trends were noted. **c** Fa-CI plots showing the synergetic effect of ETO and TMZ on GBM TSs. Through Fa-CI plots, the drug dose of ETO was determined to be 100 μM with the best synergism

The histopathological characteristics of GBM tissues, from which TSs were derived, are described in Additional file 1: Table S2. To confirm the effects of ETO and/or TMZ on GBM TSs, their cytotoxicity was evaluated using ATP and WST assays. Three out of the four GBM TSs showed significantly lowered ATP production rates in the combination treatment group compared to the control group (Fig. 2a). A similar trend was noted in cell viability results (Fig. 2b). Fa-CI plots showed a synergistic effect (CI < 1) of ETO and TMZ combination therapy (Fig. 2c).

### Combined treatment reduces bioenergetics and increases apoptosis

**Fig. 3:**
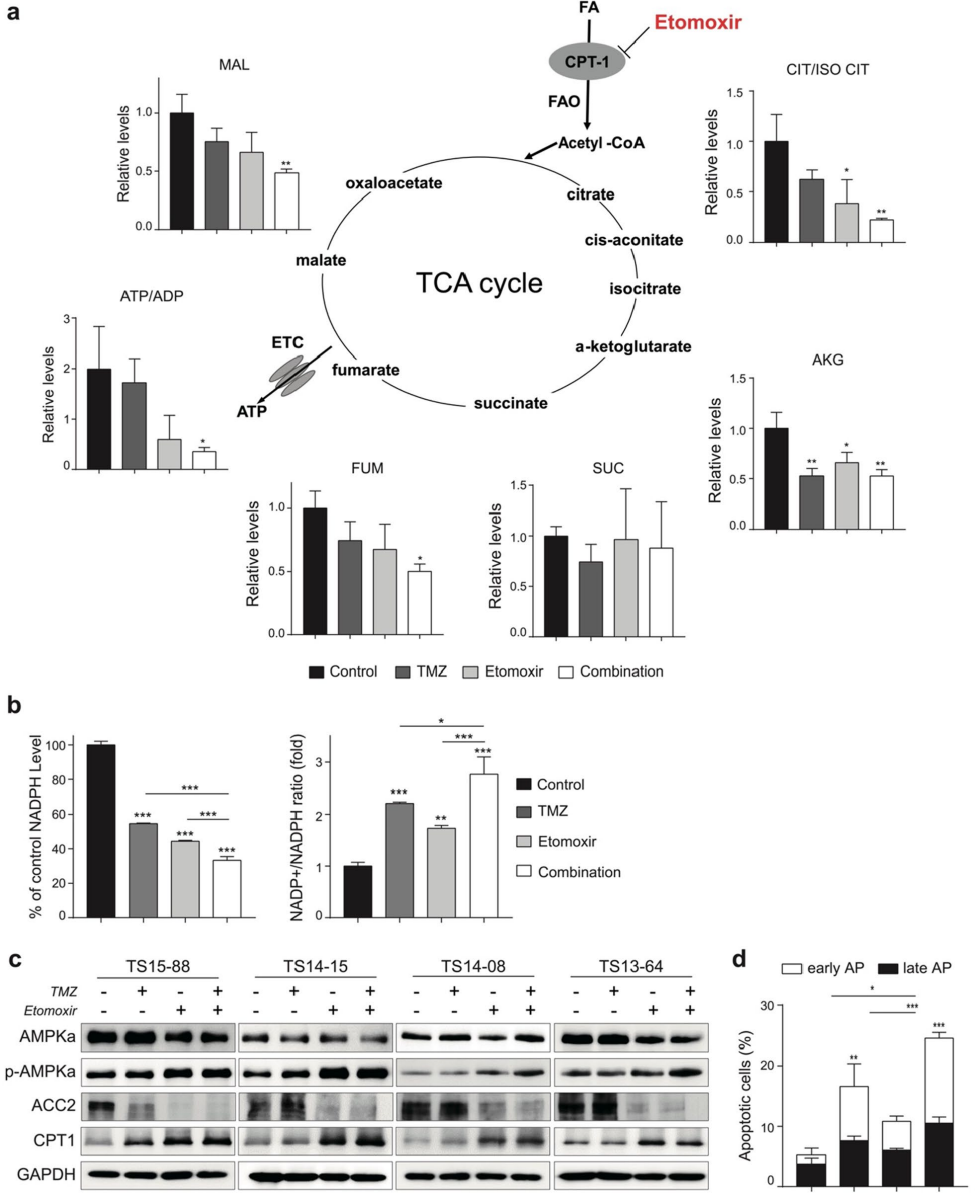
Treatment with ETO and/or TMZ suppresses energy metabolism. **a** The TCA cycle intermediates were quantitatively measured by subjecting TS13-64 treated with ETO and/or TMZ to LC/MS analysis. Relative levels of each intermediate compared to the non-treated control TSs were calculated. All intermediates were decreased in ETO and/or TMZ treatment, but combination treatment outstands others in ATP production. **b** The NADPH level and NADP^+^/NADPH ratio were calculated based on the non-treated control group. The pattern of decreasing NADPH and increasing NADP^+^ supports that ATP production via mitochondrial respiration is most inhibited by combination treatment. **c** The expressions of FAO regulatory proteins in GBM TSs were estimated by western blots analysis. The AMPK-ACC2-CPT1 pathway were prominent in ETO and combination groups. **d** Apoptotic cells were calculated as the percentage compared to the initial cell number. Significantly increased apoptosis was observed in the combination group

To further understand the impact of ETO and TMZ combination therapy on mitochondrial functions, the levels of TCA cycle intermediates, ATP/ADP ratio, NADP^+^/NADPH ratio, and OCR were calculated in TS13-64. Groups treated with ETO or TMZ alone exhibited decreased levels of these intermediates compared to the control group, but the difference was not statistically significant; however, the combination group showed an overall diminution with a significant decrease in ATP production (Fig. 3a). The decrease in the isocitrate level affected the mitochondrial NADPH formation, which was confirmed by the increased NADP^+^/NADPH ratio (Fig. 3b). The OCR decreased with ETO alone treatment; however, no significant effect was observed in response to combination treatment (Additional file 1: Fig. S1).

To evaluate the role of the AMPK-ACC2-CPT1 pathway in restoring FAO, the related protein expressions were confirmed by performing western blot analysis. In activated AMPK pathway, decreased ATP level facilitated AMPKα and ACC2 phosphorylation thereby promoting CPT1 availability. ETO alone and combination treatments showed this reaction. Interestingly, CPT1 production itself increased significantly, which was not supposed to be related to the AMPK pathway(Fig. 3c). When compared to the individual treatment groups, apoptosis was considerably higher in the combination group (Fig. 3d). These results suggest that the combined treatment of ETO and TMZ significantly diminishes bioenergetics and increases apoptosis.

### Combined treatment with ETO and TMZ suppresses the stemness and invasiveness of GBM TSs

**Fig. 4:**
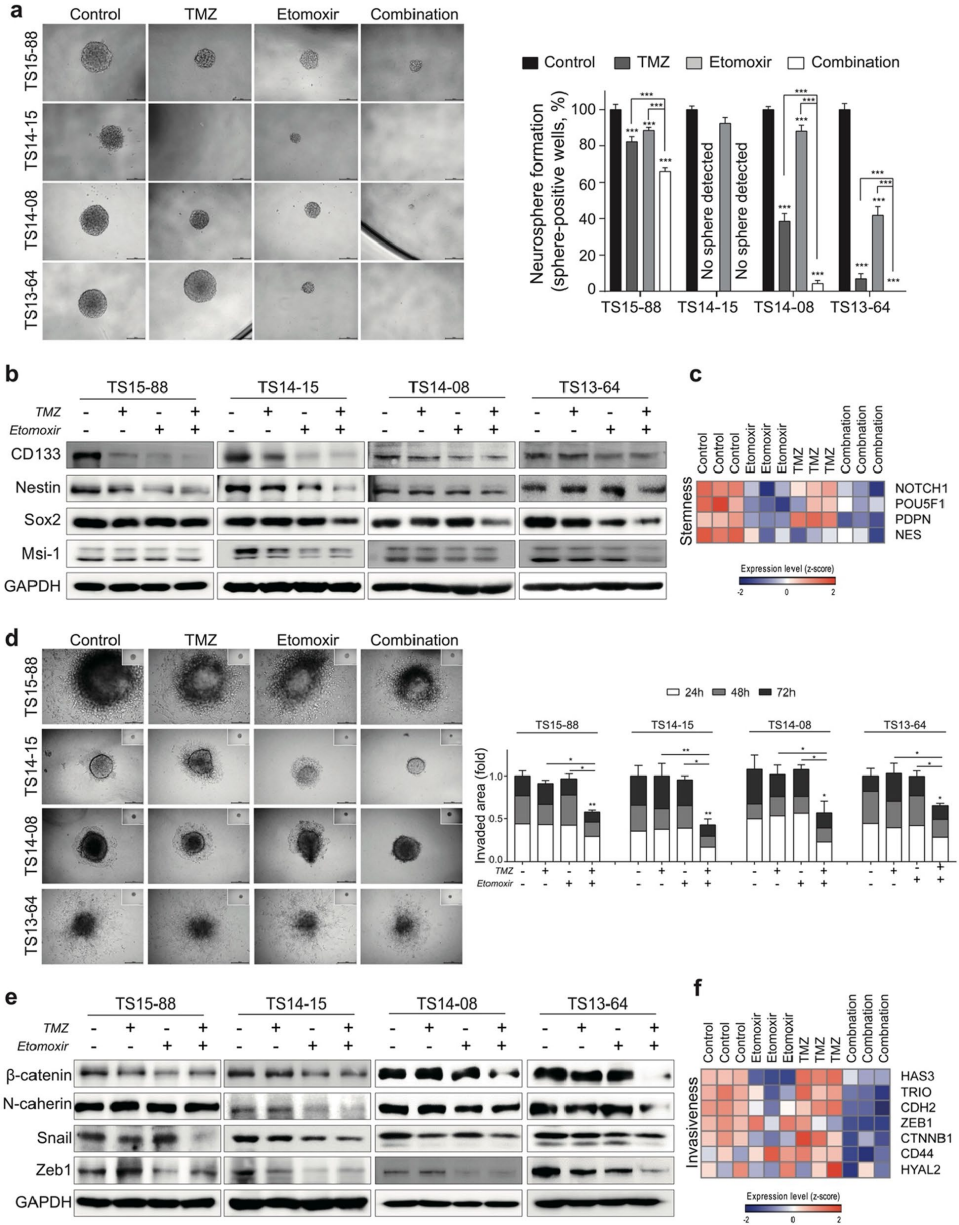
Evaluation of stemness and invasiveness after treatment with ETO and TMZ. **a** Neurosphere formation analysis was performed to detect the stemness of spheroid-shaped GBM TSs, which was measured as the percentage of sphere positive wells. TS 14–15 was the only O(6)-methylguanine-DNA methyltransferase (MGMT) methylated GBM TS in this study and presented dramatical suppression on TMZ, thereby hard to judge the combination effect. In other three TSs, combined treatment significantly inhibited neurosphere formation and its growth compared to others. **b** The expression of proteins related to stemness was measured by performing western blot analysis. **c** The heat map depicted the expression of stemness-related genes. Downregulated gene expression seemed to be more related with ETO and combination treatment. **d** The invasiveness of implanted GBM TSs treated with ETO and TMZ was confirmed by performing 3D collagen matrix invasion experiment. Invaded areas were significantly decreased in combination treatment, and this trend continues by the time. **e** Western blot analysis was performed to measure proteins related to epithelial-mesenchymal transition (EMT) and invasiveness. **f** The heat map depicted the expression of invasiveness-associated genes. Downregulation seemed to be more related with ETO and combination treatment

A neurosphere formation assay was conducted to determine the effect of individual or combined treatment with ETO and TMZ on stemness. Combined treatment with ETO and TMZ significantly suppressed the neurosphere formation capacity of TS15-88, TS14-08, and TS13-64 compared to the individual treatment groups. TS 14–15 was the only O(6)-methylguanine-DNA methyltransferase (MGMT) methylated GBM TS in this study and presented dramatical suppression on TMZ, thereby hard to judge the combination effect (Fig. 4a). The extreme limiting dilution analysis (ELDA) was performed additionally to increase the reliability of the experiment, and it was confirmed that the complex group lowered the stem cell frequency in two GBM TSs cells (Additional file 1: Fig. S2). The stemness-related proteins such as CD133, Nestin, Sox2, PDPN and Mis-1 were decreased (Fig. 4b) and associated genes such as *NOTCH1*, *POU5F1*, *PDPN* and *NES* were downregulated in the ETO alone as well as combination treatment groups (Fig. 4c). The combination treatment inhibited matrix invasion more than other groups (Fig. 4d). EMT markers such as β-catenin, N-cadherin, Snail and Zeb1, which are related to invasiveness, also significantly decreased in the combination group (Fig. 4e). The expression of relative genes such as *HAS3*, *TRIO*, *CDH2*, *ZEB1*, *CTNNB1*, *CD44* and *HYAL2* further decreased in the combination group (Fig. 4f).

### Transcriptional profile changes in response to combination treatment

When comparing the top 30% of differentially expressed genes (DEGs) to those in controls, hierarchical clustering revealed substantial intragroup clustering and unique expression patterns (Additional file 1: Fig. S3a). Functional annotations using the Gene Ontology (GO) database revealed downregulation of anabolism, mitosis, and cell proliferation. In contrast, an upregulation was noted in the catabolic process and intrinsic apoptotic signaling pathway via endoplasmic reticulum stress following combined therapy (Additional file 1: Fig. S3b, c).

### Therapeutic responses in mouse orthotopic xenograft models

**Fig. 5:**
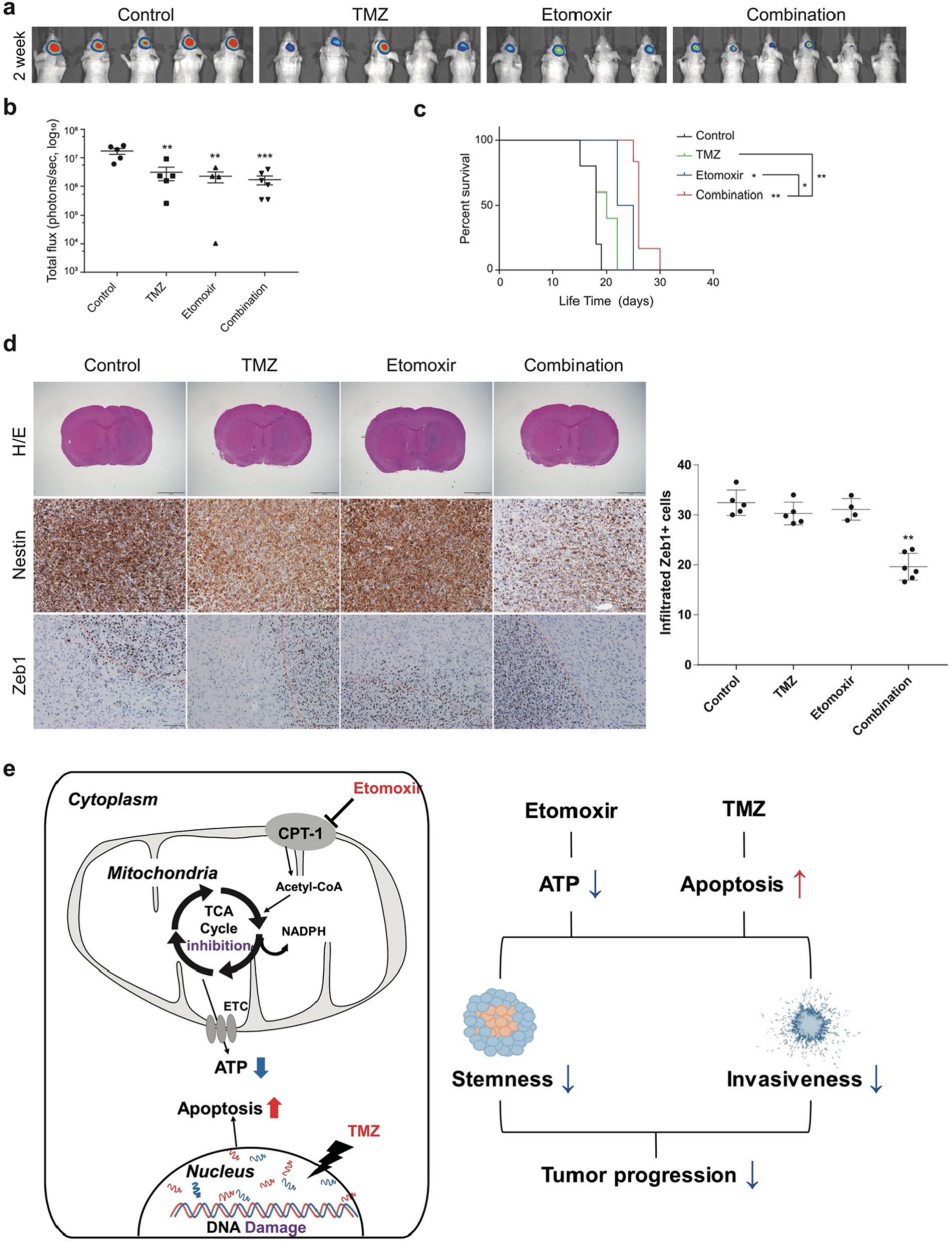
Therapeutic responses in mouse orthotopic xenograft models. **a** Bioluminescence imaging was used to assess tumor volumes as flux. **b** The total flux from the region of interest (ROI) drawn on the mouse brain was measured using an In Vivo Imaging System (IVIS), showing the lowest level in combination treatment group. **c** The Kaplan–Meier survival curve showed increased survival of mice treated with the combination of ETO and TMZ compared to the control. **d** Hematoxylin & Eosin (H&E) stain (magnification × 1.25) and Immunohistochemistry (IHC) (magnification × 20) was done for the sacrificed mice brains. Zeb1-positive cells were counted and compared among different treatment groups (outside the red line in d). **e** Graphic summary of the article. ETO inhibits bioenergetics and decreases ATP production, while TMZ promotes DNA damage and induces apoptosis. Combining these two drugs with different mechanism, synergistically reduce the stemness and invasiveness of the tumor cells, thereby inhibiting tumor progression as a consequence

TS 13–64 was selected because of its aggressive behavior in mouse models. Tumor growth and survival were evaluated in response to individual as well as combined treatment. Bioluminescence imaging showed that tumor growth was more suppressed in the combination group compared to the individual treatment groups (Fig. 5a). All groups started with 6 mice, but some individuals were excluded from the experiment due to surgical failure. Several mice of control and TMZ alone group were expired right after the IVIS imaging. There was a significant difference in total flux, especially in the combination group (Fig. 5b) compared to the group subjected to individual treatments. In the Kaplan–Meier survival analysis, each treatment group exhibited differing survival rates, and the combination group had a superior outcome (Fig. 5c, Additional file 1: Fig. S4). IHC staining revealed that the expression of Nestin, a stem cell marker, was downregulated in combined treatment. In addition, the combined therapy was more effective than the individual treatments in reducing the number of Zeb1-positive cells, which are indicators of invasive cells, outside the gross tumor mass (Fig. 5d) (Additional file 2).

## Discussion

Despite their rarity [31], cancer stem cells (CSCs) are believed to play an essential role in drug resistance in refractory tumors [32]. CSCs are known to adapt distinct metabolic profiles [33], and this metabolic reprogramming has been highlighted as a potential therapeutic target [34]. The Warburg effect, which is an anaerobic glycolysis model [35], has been criticized for its bioenergetic inefficiency. Recent studies have shown that numerous metabolic pathways are upregulated together in cancer [36, 37]. Among them, FAO is known to be related to stress conditions that require increased ATP supply, such as metastasis [38] and recurrence [39]. Recently, Berge et al. [40] demonstrated that FAO inhibition is associated with the suppressed proliferation of glioma cells. Kant et al. [41] further showed that FAO is a key metabolic route in GBM, allowing the tumor to adapt to its changing environment via metabolic flexibility. Therefore, we hypothesized that GBM depends on the catabolic FAO pathway and that its inhibition has a significant effect on tumor growth and survival. Since previous studies focused on FAO inhibitors alone but not combined with TMZ, we tried to investigate their combination therapeutic effects.

Before designing a detailed study, we analyzed our clinical data to assess whether FAO was upregulated in GBM. Tumor-free cortex tissues from various glioma patients and GBM IDH-wildtype specimens were collected during surgeries performed at our hospital. Gene sets related to cancer metabolism were compared through RNA-sequencing, and it was confirmed that the expression of FAO-related genes, especially *CPT1A*, was significantly upregulated in GBM. Since previous studies primarily focused on identifying single effect of new anticancer drugs, we analyzed whether FAO inhibitors could improve the outcomes of current treatment modalities. Patient-derived GBM TSs are known to contain CSCs and exhibit actual tumor behaviors; therefore, they are considered a good platform to test CSC-targeting agents [42]. In this study, we treated four patient-derived GBM TSs and xenograft mouse models with individual or combination therapy using TMZ and ETO.

The results of the combination treatment were significantly better than those of the individual treatments. The combined treatment with ETO and TMZ reduced ATP production and cell viability in GBM TSs; moreover, they exhibited a synergetic effect (Fig. 2). TMZ induces DNA methylation damage [43], while ETO inhibits CPT1/FAO responsible for ATP generation, and according to our results, the percentage of apoptotic cells in GBM TSs increased when TMZ alone was treated, moreover, combined treatment with etomoxir further increased percentage of apoptotic cell (Fig. 3d). Although their mechanisms of action are entirely different, their synergism could be due to the common consequence of inhibiting cell growth (Fig. 5).

The TCA cycle generates the reducing equivalent NADPH, which is a potent non-enzymatic antioxidant. It helps in ATP generation via oxidative as well as substrate-level phosphorylation [44]. Pike et al. [16] reported that FAO inhibition by ETO decreased the influx of fatty acids into the mitochondria and decreased NADPH and ATP levels. In our study, ETO alone and in combination with TMZ regulated the TCA cycle in GBM TSs to reduce ATP production and significantly inhibited NADPH production (Fig. 3a, b). In addition, the OCR of GBM TSs, which is associated with mitochondrial respiration, was significantly reduced in response to treatment with ETO (Additional file 1: Fig. 1).

Previous studies have shown that interruption of FAO pathway by ETO upregulates glycolysis and glutaminolysis via an anaplerotic reaction [16]. If this response is sufficient for supplementing cellular metabolism, then efforts to restore the FAO pathway may not be necessary for tumor cells. To investigate this, we compared the expression of FAO regulatory proteins in response to with and without treatment. In normal cells, induced ATP depletion due to hypoxia, nutrient deprivation or exercise, and increased AMP/ATP ratio facilitates the activation of the energy sensor AMPK. Phosphorylated AMPK inactivates ACC2 via phosphorylation, and this prevents the conversion of cytosolic acetyl-CoA to malonyl-CoA. Since malonyl-CoA had an inhibitory effect on CPT1, CPT1 availability is increased and mitochondrial fatty acid uptake is promoted [45–47]. Western blot analysis showed that thisalso occurred in GBM TSs as same as normal cells, which was especially prominent in the combination group. One unexpected result was the increased CPT1 production according to ETO treatment. It is presumed that GBM TSs upregulate CPT1 expression to combat ETO's irreversible interference with CPT1 and restore the FAO pathway (Fig. 3c). This implies that the FAO pathway plays an essential role in GBM metabolism.

The fact that high ATP production is highly correlated with tumor progression and metastasis has been known through previous studies, but the exact mechanism requires further investigations [50]. Recently, FAO has been suggested to be essential for the survival of cancer stem cells not only in providing essential energy for maintaining tumor cells but also in regulating oxidative stress and in participating in the activity of all metabolic enzymes through protein acetylation [51]. According to previous study, knockdown of CPT1 inhibited invasiveness in breast cancer cell line (MDA-MB-231) [52]**.** In another studies, etomoxir treatment reduced invasion rate of breast cancer cells [53] and decreased sphere formation rate and the CD44 expression, a stemness marker, in lung adenocarcinoma sphere cells (H460) [48]. Similarly, our results showed that the inhibition of stemness and invasiveness was induced by etomoxir treatment, an inhibitor of CPT1, and that ETO alone reduced the expression of stemness and invasiveness-related genes more effectively than TMZ monotherapy group. (Fig. 4c, f). However, this transcriptional changes seems do not lead to actual changes in stemness and invasiveness capacity of tumorspheres. TMZ alone appeared to induce less alteration in stemness-related gene expressions than ETO alone, but inhibited neurosphere formation capacity more effectively. And the experiment showed that both treatments failed to reduce invasiveness (Fig. 4a, c). On the other hand, the combined treatment further increased gene downregulation and demonstrated a significant decrease in stemness and invasiveness capacities (Fig. 4). This can be interpreted as the combined treatment overcomes the limitations of monotherapy at the tumorsphere level.

Since ETO is currently used for preclinical research only because of its hepatotoxicity [49], we note that there is a need for the improvement or development of anti-FAO agents. Also, the penetration capacity of ETO through blood brain barrier (BBB) is still unknown. Due to the above reasons and the lack of the laboratory resources, in vivo experiment in the pretreatment format was done. However, we think our study still has significance in terms of proof-of-concept. The superior results of combination therapy on stemness, invasiveness, expression regulation with related proteins and genes, and survival reveal it as a promising strategy to control the unregulated growth of GBM, which is difficult to achieve with conventional chemotherapy (Figs. 4, 5). The brief mechanism of combination therapy was presented as a graphic summary (Fig. 5e) [16, 43]. To the best of our knowledge, this is the first attempt to combine FAO inhibitors with TMZ, and we believe that these findings will contribute to better anticancer drug selection in the future.

## Conclusion

We used patient-derived tissues and GBM TSs to demonstrate that GBM upregulates the expression of FAO-related genes and is FAO-dependent for its growth. Furthermore, the treatment combining ETO with TMZ induced a decrease in ATP levels and increased apoptosis. This led to the suppression of stemness and invasiveness and a prolonged survival as observed in the mouse models (Fig. 5). These results imply that this combination strategy is a potential alternative to currently used chemotherapies. Further investigations are needed to better understand the implications of combination therapies and to identify treatments strategies.

## Supplementary Information

**Additional file 1: Table S1.** Clinicohistopathologic characteristics of the tumor-free cortex and GBM tissue. **Table S2.** Histopathologic characteristics of GBM tissues from which TSs were derived. **Figure S1.** Mitochondrial stress analysis showing decreased OCR in TS13-64 following etomoxir treatment. **Figure S2.** The sphere-forming capacity of GBM TSs cells were examined using the Extreme Limiting Dilution Assay (ELDA). TS cells were seeded in increasing order per well (1, 5, 10, 20, and 50 cells per well) in TS complete media with or without drugs in 96-well plates. The number of tumor spheres in each well was counted 7 days after plating. The online ELDA analysis tool was used (http://bioinf.wehi.edu.au/software/elda). **Figure S3.** Effects of etomoxir and TMZ combination treatment on gene expression profile as determined by RNA-sequencing. TS13-64 cells were treated with etomoxir and/or TMZ for 72 h, and RNA-sequencing was performed to determine the gene expression profiles. **a** Average linkage hierarchical clustering was achieved with Euclidean distance used as a distance metric for genes showing average expression in the top 30%. Expression was visualized as a heat map using GENE-E software. Among the DEGs between the control and combination groups, genes with **b**. upregulated and **c.** downregulated expression following combination therapy were functionally annotated, clustered, and visualized in an enrichment map. Each node represents a GO term with the size of the nodes indicating the statistical significance of over-representation. The kappa score connection is represented by an edge between two nodes. Clustered modules are represented by node colors, and the most important GO phrases for each module are shown with highlighted labels. **Figure S4.** Kaplan–Meier survival curves following etomoxir-alone, TMZ-alone, or etomoxir-TMZ combination treatment. **a**–**c** The etomoxir-only and combination groups had significantly improved survival compared to the control group, in contrast to the TMZ-only group. **d**–**e** The combination group exhibited improved survival compared to the etomoxir- or TMZ-only groups.

**Additional file 2: Figure S5**. Original image indicating AMPKa, p-AMPKa, ACC2, CPT1 and GAPDH for Western blots of figure 3c. **Figure S6**. Original image indicating CD133, Nestin, SOX2, Msi-1 and GAPDH for Western blots of figure 4b. **Figure S7.** Original image indicating CD133, Nestin, SOX2, Msi-1 and GAPDH for Western blots of figure 4e.

## Data Availability

The datasets generated during and/or analyzed during the current study are available from the corresponding author on reasonable request.
