# Supplementary material for: Etomoxir, a carnitine palmitoyltransferase 1 inhibitor, combined with temozolomide reduces stemness and invasiveness in patient-derived glioblastoma tumorspheres
Source: Cancer Cell Int. 2022 Oct 11;22:309. doi: 10.1186/s12935-022-02731-7 (PMC9552483; doi:10.1186/s12935-022-02731-7)
Supplement: Supplementary file 1 — Additional file 1: Table S1. Clinicohistopathologic characteristics of the tumor-free cortex and GBM tissue. Table S2. Histopathologic characteristics of GBM tissues from which TSs were derived. Figure S1. Mitochondrial stress analysis showing decreased OCR in TS13-64 following etomoxir treatment. Figure S2. The sphere-forming capacity of GBM TSs cells were examined using the Extreme Limiting Dilution Assay (ELDA). TS cells were seeded in increasing order per well (1, 5, 10, 20, and 50 cells per well) in TS complete media with or without drugs in 96-well plates. The number of tumor spheres in each well was counted 7 days after plating. The online ELDA analysis tool was used (http://bioinf.wehi.edu.au/software/elda). Figure S3. Effects of etomoxir and TMZ combination treatment on gene expression profile as determined by RNA-sequencing. TS13-64 cells were treated with etomoxir and/or TMZ for 72 h, and RNA-sequencing was performed to determine the gene expression profiles. a Average linkage hierarchical clustering was achieved with Euclidean distance used as a distance metric for genes showing average expression in the top 30%. Expression was visualized as a heat map using GENE-E software. Among the DEGs between the control and combination groups, genes with b. upregulated and c. downregulated expression following combination therapy were functionally annotated, clustered, and visualized in an enrichment map. Each node represents a GO term with the size of the nodes indicating the statistical significance of over-representation. The kappa score connection is represented by an edge between two nodes. Clustered modules are represented by node colors, and the most important GO phrases for each module are shown with highlighted labels. Figure S4. Kaplan–Meier survival curves following etomoxir-alone, TMZ-alone, or etomoxir-TMZ combination treatment. a–c The etomoxir-only and combination groups had significantly improved survival compared to the control group, in contrast t [file 12935_2022_2731_MOESM1_ESM.docx]

**Supplementary Table S1. Clinicohistopathologic characteristics of the tumor-free cortex and GBM tissue**

|  |  | GBM tissue (n=112) | Tumor-free cortex  (n=35) |
| --- | --- | --- | --- |
| Age |  | 57.98 | 51.83 |
| Sex |  |  |  |
|  | Female | 43 (38.39%) | 17 (48.57%) |
|  | Male | 69 (61.61%) | 18 (51.43%) |
| Diagnosis* | |  |  |
|  | GBM | 112 (100%) | 22 (62.86%) |
|  | Others | 0 (0%) | 11 (31.43%) |
| IDH status | |  |  |
|  | wild-type | 112 (100%) |  |
|  | mutant | 0 (0%) |  |
| MGMT status | |  |  |
|  | Methylated | 38 (33.93%) |  |
|  | Unmethylated | 74 (66.07%) |  |
| 1p/19q |  |  |  |
|  | intact | 106 (94.64%) |  |
|  | 1p LOH only | 2 (1.76%) |  |
|  | 19q LOH only | 1 (0.89%) |  |
|  | co-deletion | 1 (0.89%) |  |
| TERT mutation | |  |  |
|  | C250T | 12 (10.71%) |  |
|  | C228T | 59 (52.68%) |  |
|  | No mutation | 17 (15.18%) |  |

*** All GBM tissues were obtained from patients with IDH-wildtype GBM. Tumor-free cortex tissues were obtained from various patients including other glioma cases.**

**Supplementary Table S2. Histopathologic characteristics of GBM tissues from which TSs were derived**

| **Tumorsphere** | **Tissue pathology** | ***MGMT***  **promoter** | **Codeletion of**  **1p/19q** | **Verhaak’s subtype of tissue**^1^ | | **Prognostic subtype of tissue**^2^ |
| --- | --- | --- | --- | --- | --- | --- |
| TS15-88 | Glioblastoma,  *IDH1*_wild type | Unmethylated | Intact/Intact | Mesenchymal | Invasive | |
| TS14-15 | Glioblastoma,  *IDH1*_wild type | Methylated | Intact/LOH | Classical | Invasive | |
| TS14-08 | Glioblastoma,  *IDH1*_wild type | Unmethylated | Intact/Intact | Mesenchymal | Invasive | |
| TS13-64 | Glioblastoma,  *IDH1*_wild type | Unmethylated | Intact/Intact | Classical | Invasive | |

Abbreviations: IDH, isodehydrogenase; WT, wildtype; Codeletion of 1p/19q, the presence of codeletion of chromosome 1p/19q; MGMT, O-6-methylguanine-DNA methyltransferase; EGFR, epidermal growth factor receptor

**References of Supplementary Table S2**

1. Verhaak RGW, Hoadley KA, Purdom E, Wang V, Qi Y, Wilkerson MD, Miller CR, Ding L, Golub T, Mesirov JP, Alexe G. (2010) Integrated genomic analysis identifies clinically relevant subtypes of glioblastoma characterized by abnormalities in PDGFRA, IDH1, EGFR, and NF1. *Cancer Cell*. 17(1):98-110. https://doi.org/10.1016/j.ccr.2009.12.020

2. Park J, Shim JK, Yoon SJ, Kim SH, Chang JH, Kang SG. (2019) Transcriptome profiling-based identification of prognostic subtypes and multi-omics signatures of glioblastoma. *Scientific Reports*. 9:10555. https://doi.org/10.1038/s41598-019-47066-y


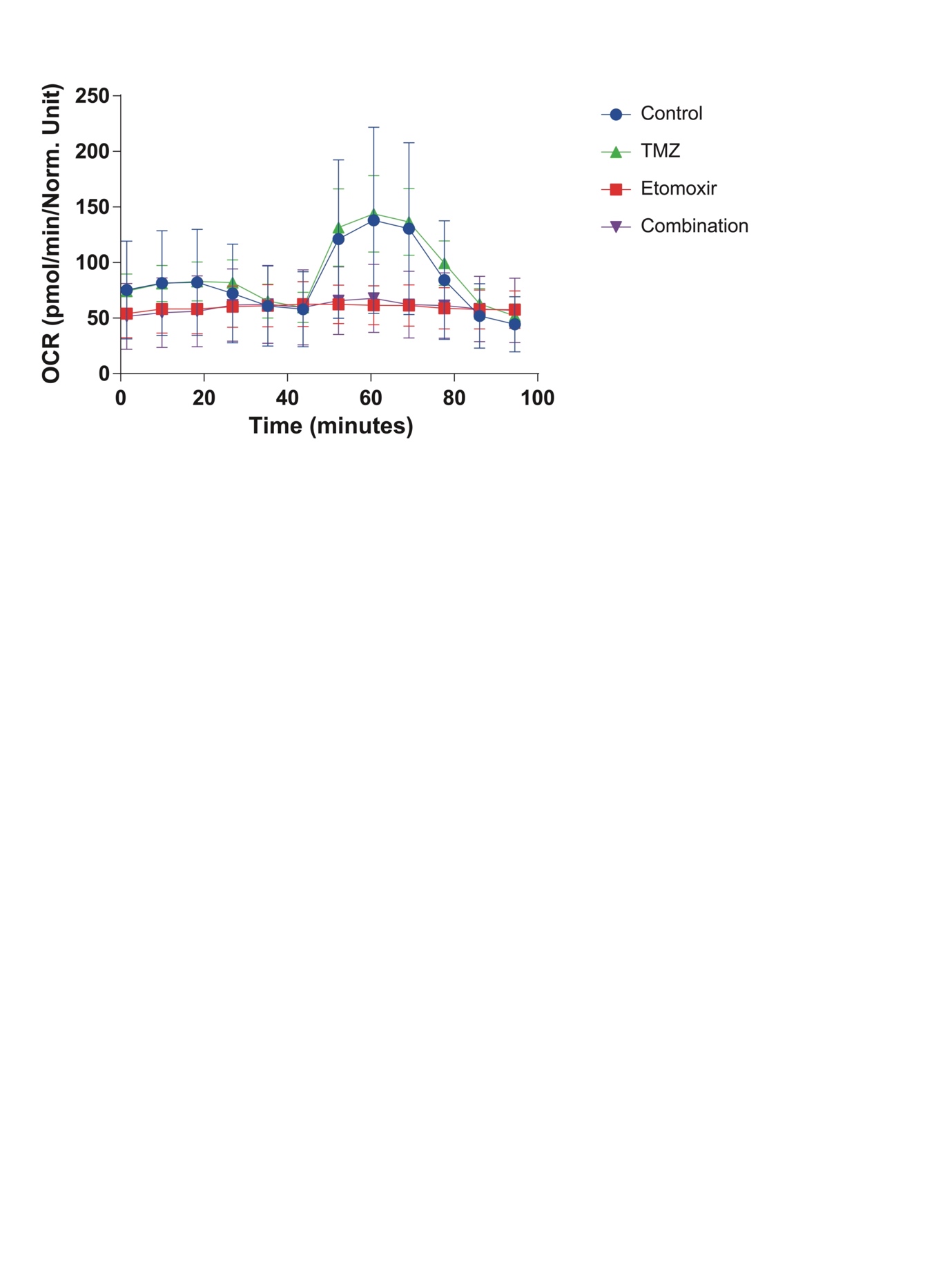


**Supplementary Fig. 1.** Mitochondrial stress analysis showing decreased OCR in TS13-64 following etomoxir treatment


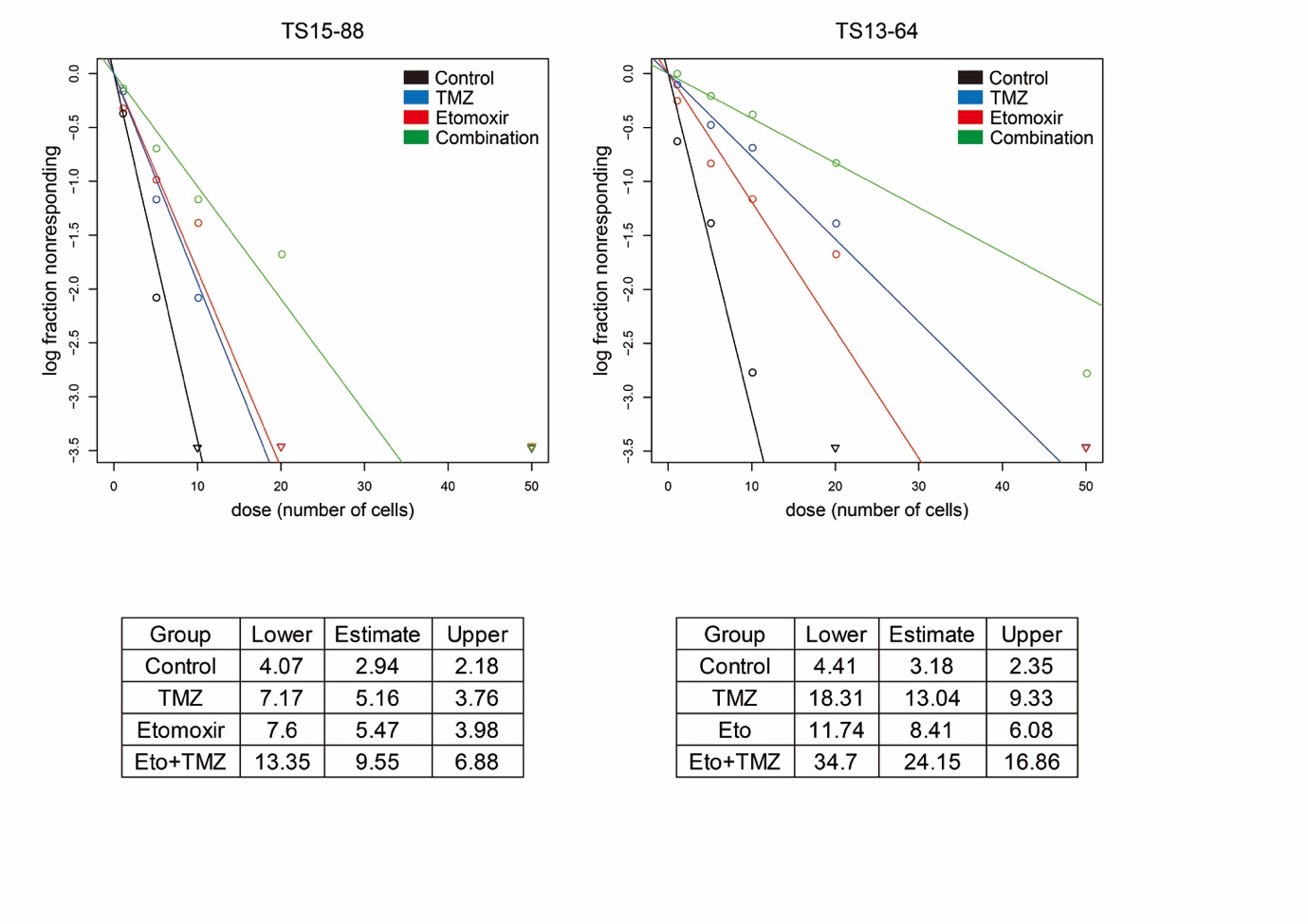


**Supplementary Fig. 2.** The sphere-forming capacity of GBM TSs cells were examined using the Extreme Limiting Dilution Assay (ELDA). TS cells were seeded in increasing order per well (1, 5, 10, 20, and 50 cells per well) in TS complete media with or without drugs in 96-well plates . The number of tumor spheres in each well was counted 7 days after plating. The online ELDA analysis tool was used (<http://bioinf.wehi.edu.au/software/elda>).

**References of Supplementary Fig. 2.**

1. Hu, Y.F. & Smyth, G.K. ELDA: Extreme limiting dilution analysis for comparing depleted and enriched populations in stem cell and other assays. J Immunol Methods 2009; 347, 70-78.


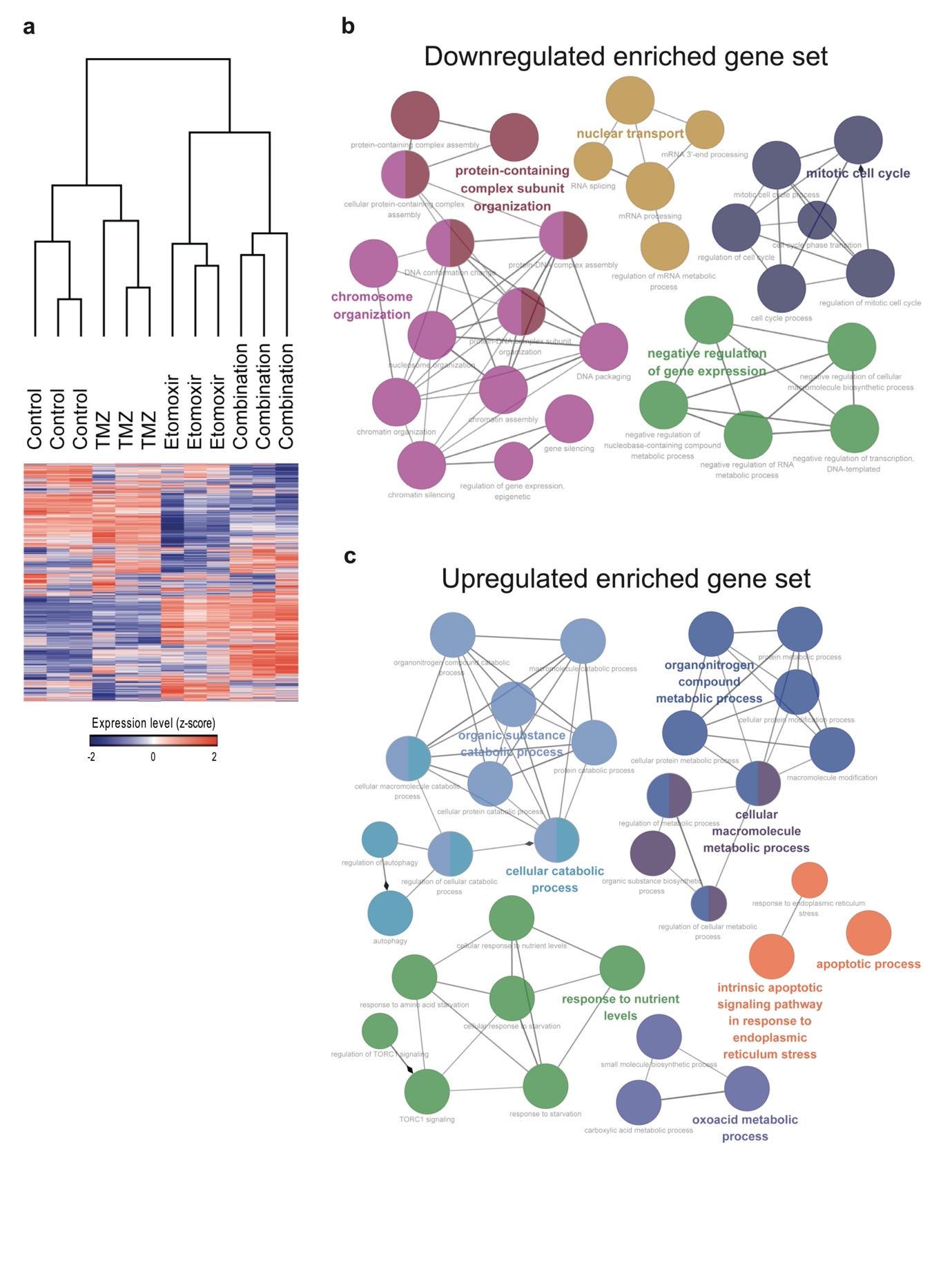


**Supplementary Fig. 3.** Effects of etomoxir and TMZ combination treatment on gene expression profile as determined by RNA-sequencing. TS13-64 cells were treated with etomoxir and/or TMZ for 72 h, and RNA-sequencing was performed to determine the gene expression profiles. **a.** Average linkage hierarchical clustering was achieved with Euclidean distance used as a distance metric for genes showing average expression in the top 30%. Expression was visualized as a heat map using GENE-E software. Among the DEGs between the control and combination groups, genes with **b.** upregulated and **c.** downregulated expression following combination therapy were functionally annotated, clustered, and visualized in an enrichment map. Each node represents a GO term with the size of the nodes indicating the statistical significance of over-representation. The kappa score connection is represented by an edge between two nodes. Clustered modules are represented by node colors, and the most important GO phrases for each module are shown with highlighted labels.


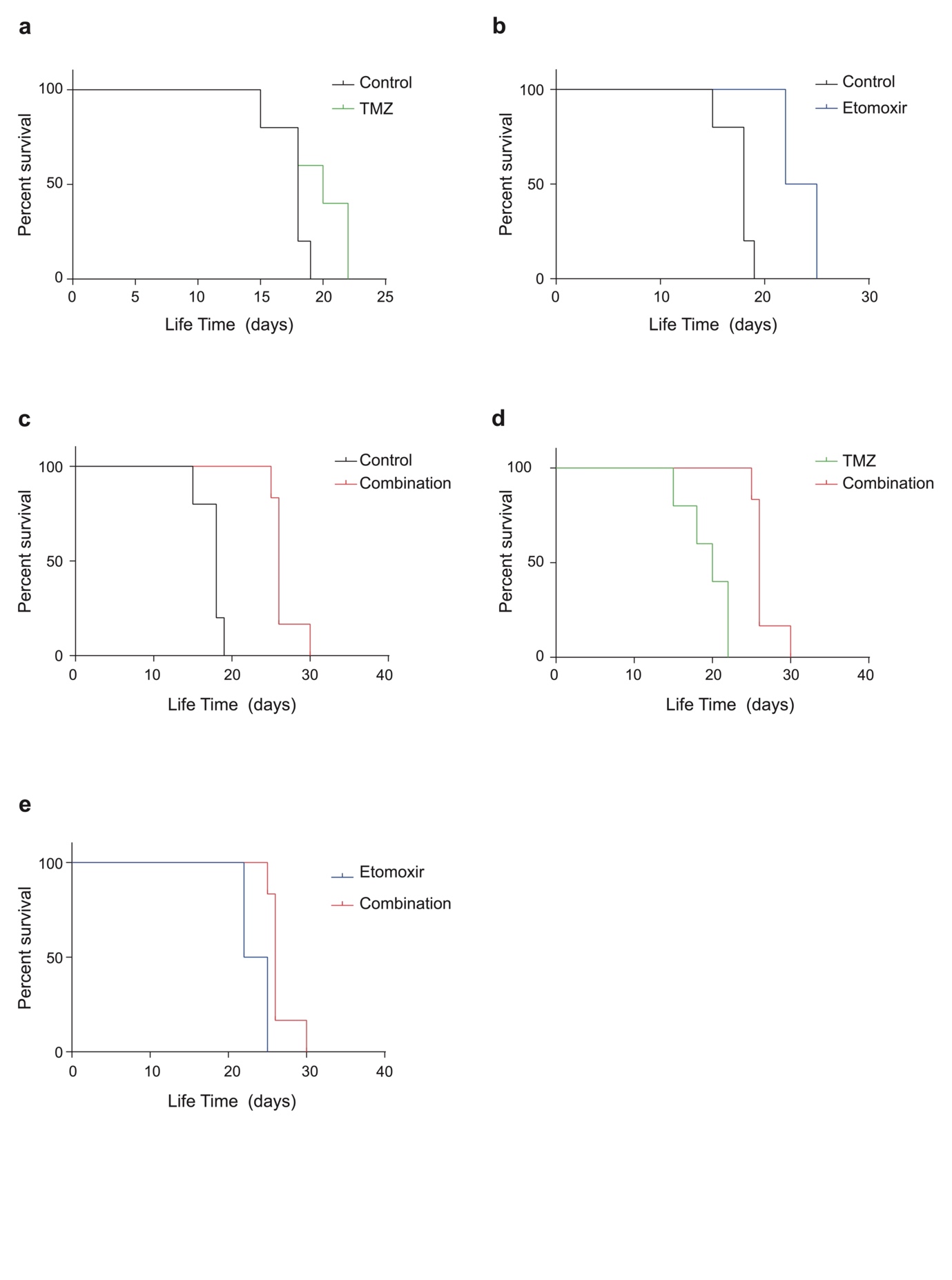


**Supplementary Fig. 4.** Kaplan-Meier survival curves following etomoxir-alone, TMZ-alone, or etomoxir-TMZ combination treatment. **a-c.** The etomoxir-only and combination groups had significantly improved survival compared to the control group, in contrast to the TMZ-only group. **d-e.** The combination group exhibited improved survival compared to the etomoxir- or TMZ-only groups.
