# Supplementary material for: Etomoxir, a carnitine palmitoyltransferase 1 inhibitor, combined with temozolomide reduces stemness and invasiveness in patient-derived glioblastoma tumorspheres
Source: Cancer Cell Int. 2022 Oct 11;22:309. doi: 10.1186/s12935-022-02731-7 (PMC9552483; doi:10.1186/s12935-022-02731-7)

**Figure S5. Original image indicating AMPKa, p-AMPKa, ACC2, CPT1 and GAPDH for Western blots of figure 3c.**


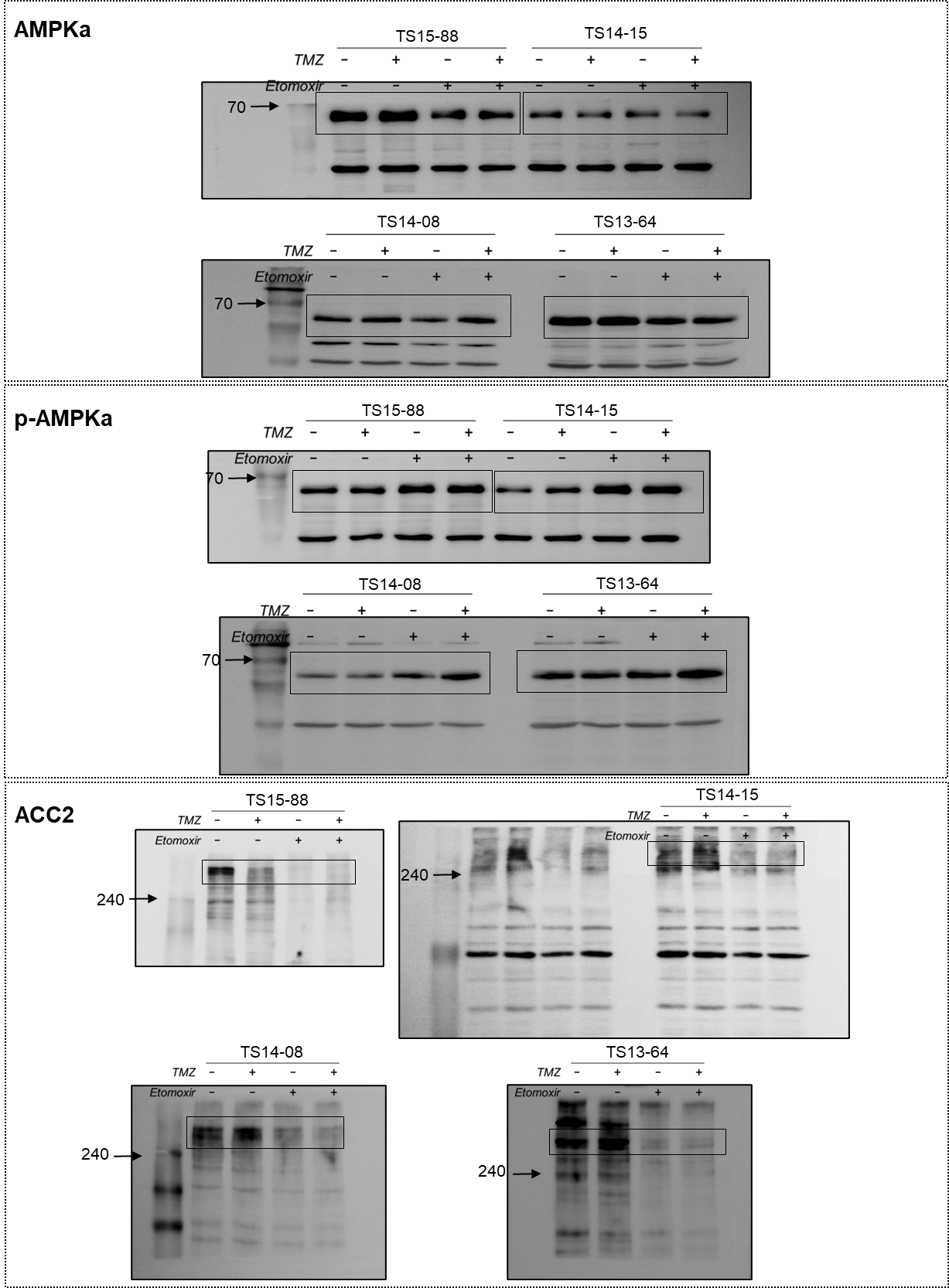

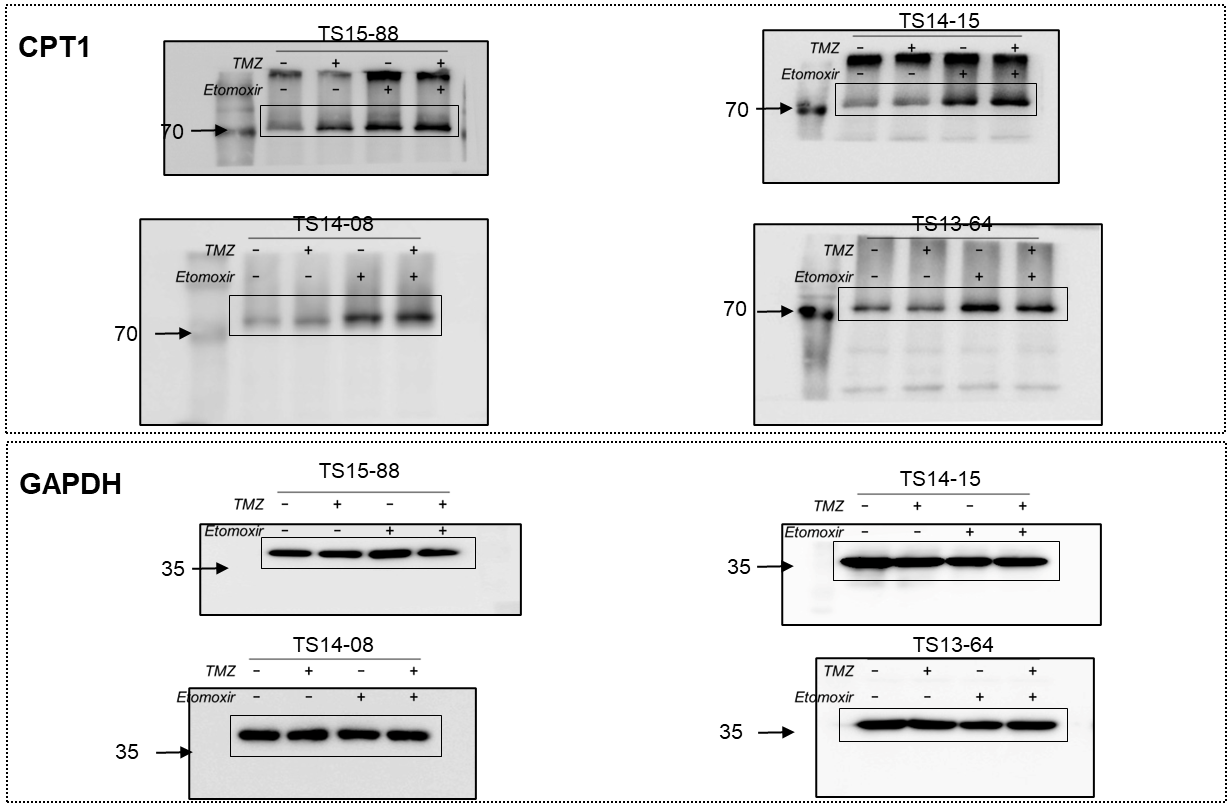


**Figure S6. Original image indicating CD133, Nestin, SOX2, Msi-1 and GAPDH for Western blots of figure 4b**


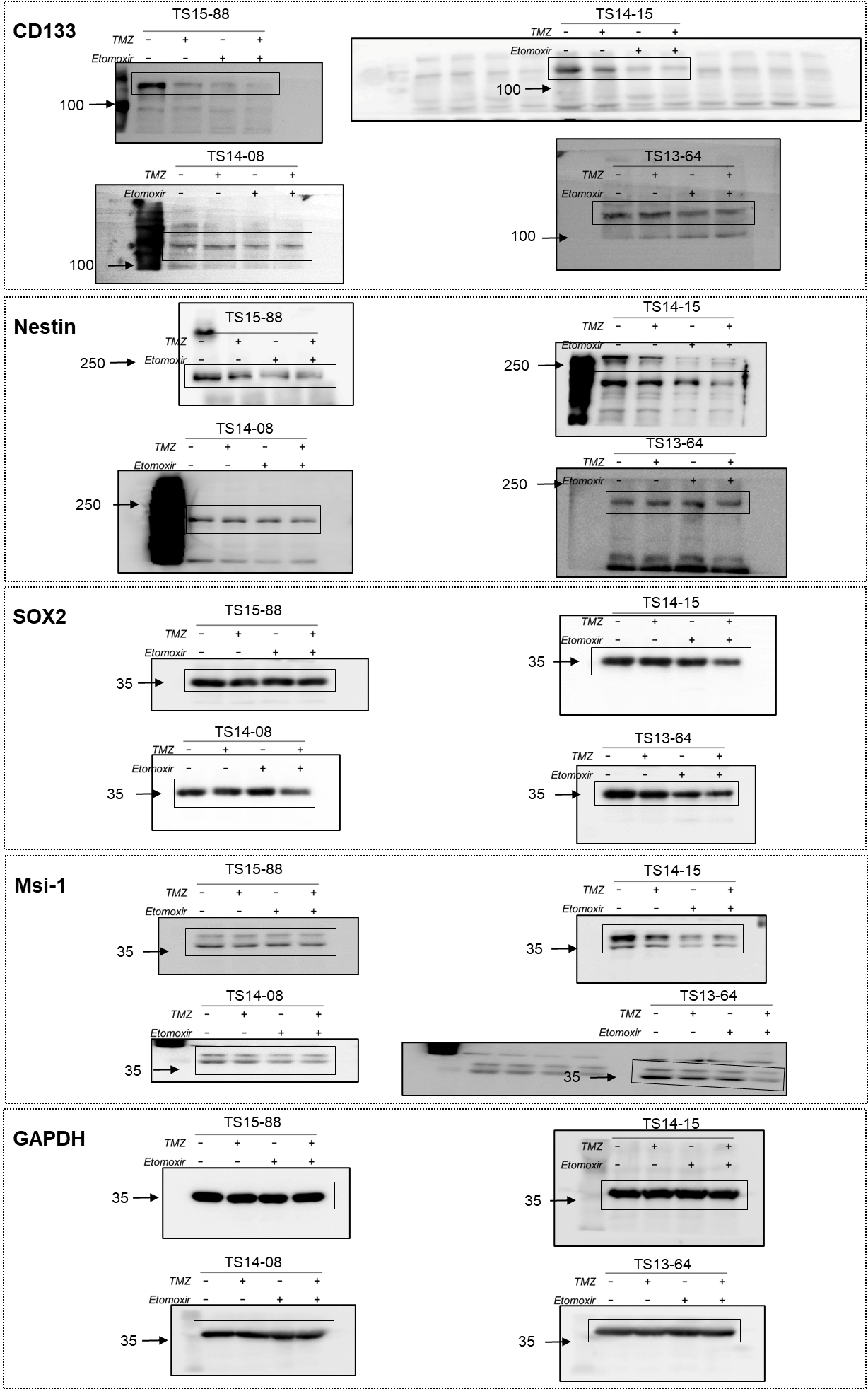


**Figure S7. Original image indicating CD133, Nestin, SOX2, Msi-1 and GAPDH for Western blots of figure 4e.**


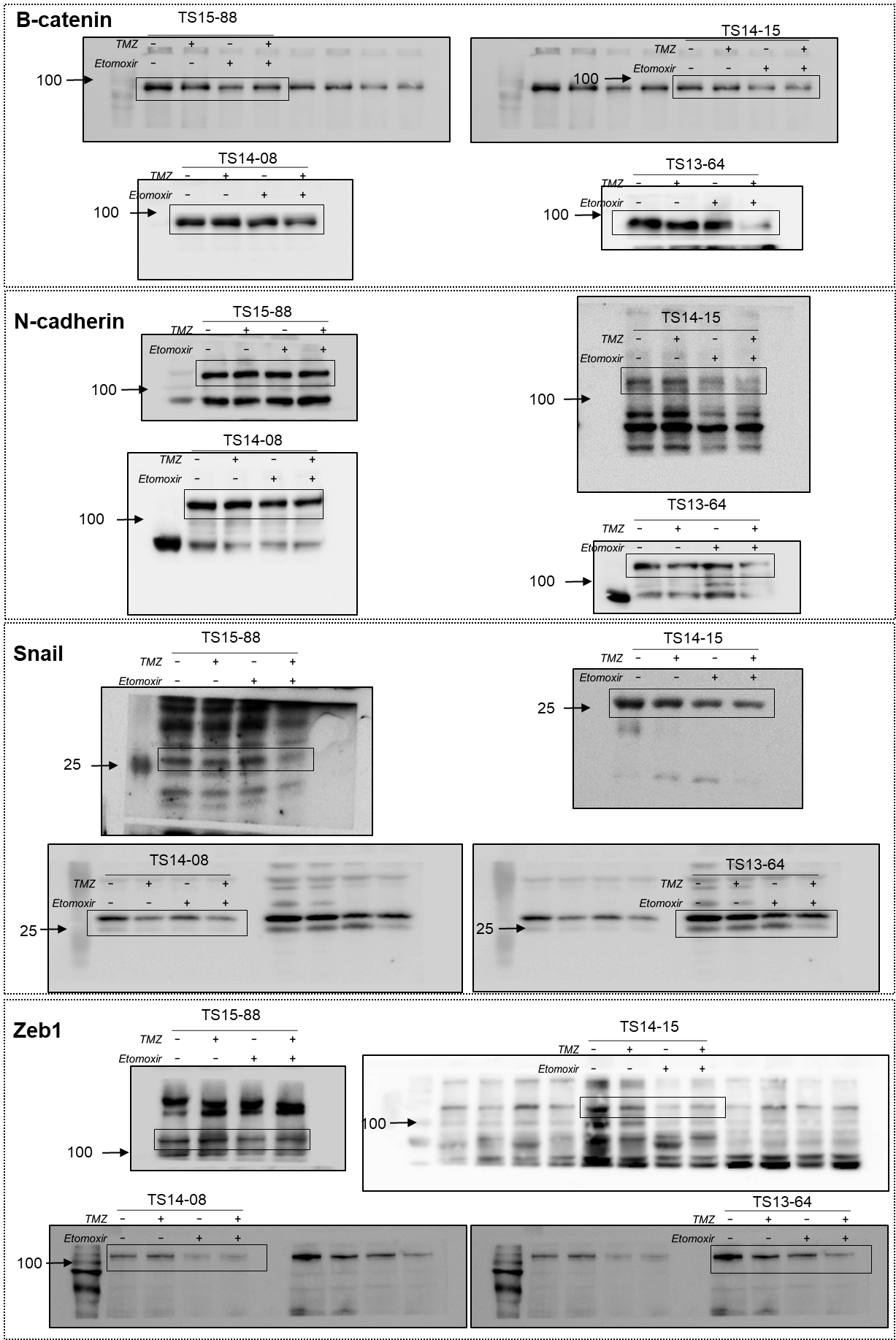

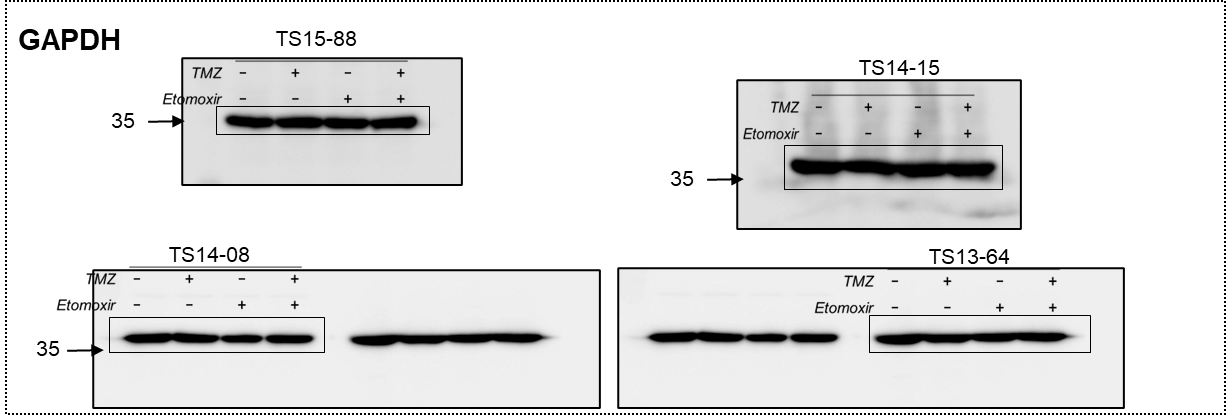

Supplement: Supplementary file 2 — Additional file 2: Figure S5. Original image indicating AMPKa, p-AMPKa, ACC2, CPT1 and GAPDH for Western blots of figure 3c. Figure S6. Original image indicating CD133, Nestin, SOX2, Msi-1 and GAPDH for Western blots of figure 4b. Figure S7. Original image indicating CD133, Nestin, SOX2, Msi-1 and GAPDH for Western blots of figure 4e. [file 12935_2022_2731_MOESM2_ESM.docx]
